# Proteomic and immunoassay characterization of a new food allergen from hazelnut (corylus avellana)

**DOI:** 10.1186/2045-7022-3-S3-P40

**Published:** 2013-07-25

**Authors:** G Picariello, R Nocerino, C Nitride, G Mamone, R Troncone, P Ferranti, R Berni Canani

**Affiliations:** 1 Institute of Food Sciences – CNR, Avellino, Italy; 2 Department of Pediatrics, University of Naples "Federico II", Naples, Italy; 3 Department of Food Science, University of Naples “Federico II”, Portici (Naples), Italy; 4 Department of Pediatrics and European Laboratory for the Investigation of Food Induced Diseases (ELFID), University of Naples “Federico II”, Naples, Italy

## Background

Hazelnut (*Corylus avellana*) is a common cause of lifetime lasting IgE-mediated food allergy. Symptoms range from mild oral allergy syndrome to severe life-threatening anaphylaxis. We aimed to identify allergenic determinants in children living in the Campania region (Italy) with hazelnut allergy.

## Methods

Otherwise healthy children with oral food challenge confirmed hazelnut allergy were prospectively evaluated. Crude protein extracts were obtained from 5 hazelnut varieties, including autochthon, Northern Italy and Oregon (USA) cultivars, with phosphate saline buffer, pH 7.2. The immunoreactive protein components were identified by SDS-PAGE electrophoresis and Western immunoblotting, using patients sera as source of specific IgE. The IgE-binding protein bands were characterized by advanced proteomic strategies and tandem mass spectrometry (MS)-based *de novo* peptide sequencing.

## Results

Four subjects were evaluated (2 male, 50%; mean age 39 m). Symptoms were: urticaria (2), angioedema (3), anaphylaxis (2). No significant differences were observed considering the main demographic and clinical characteristics at diagnosis. All children’s sera were immunoreactive to a protein, not previously annotated in database, occurring in hazelnut regardless the variety. The allergen was isolated by combined chromatographic strategies. Only one patient exhibited an additional reactivity to the vicilin-like 7S 48 kDa glycoprotein (Cor a 11). The MS-based characterization provided evidence of a high homology degree between the IgE-binding protein subunit and 11S globulin-like storage proteins expressed in other seeds. The new allergen shares structural traits with the hazelnut 11S globulin-like proteins (Cor a 9) such as the disulfide linkage of two subunits, an acidic (~35 kDa) and an alkaline (~21 kDa) one. Interestingly, only the alkaline subunit exhibits antigenic properties.

## Conclusion

A previously unrecognized hazelnut allergen was identified. Except for a faint IgE reactivity of Cor a 11 recorded in a single case, the new allergen was the unique IgE-binding protein in our patients. Future study are warranted to better define possible prognostic and immunotherapeutic implications.

## Disclosure of interest

None declared.

